# Professional self-concept and burnout among medical school faculty in South Korea: a cross-sectional study

**DOI:** 10.1186/s12909-019-1682-z

**Published:** 2019-07-05

**Authors:** Jihye Yu, Sukyung Lee, Miran Kim, Kiyoung Lim, Kihong Chang, Sujin Chae

**Affiliations:** 1 Office of Medical Education, Ajou University, Suwon, South Korea; 2 Ajou Center for Clinical Excellence, Ajou University, Suwon, South Korea; 3 Department of Psychiatry, Ajou University, Suwon, South Korea; 4 Department of Medical Education, Catholic Kwandong University College of Medicine, Incheon, South Korea

**Keywords:** Burnout, Faculty development, Faculty of medicine, Professional self-concept

## Abstract

**Background:**

Medical school faculty members have been reported to be highly likely to suffer burnout. Research is being conducted on professional self-concepts as a factor that relieves burnout in many professions. However, there is a paucity of data on the relationship between professional self-concept and burnout among medical school faculty. Professional self-concept means a perception of oneself as a member of the profession. It influences an individual’s thoughts, actions, and emotions. The more positive the professional self-concept, the higher is the self-esteem in the profession, which can contribute to reducing burnout. This study aimed to investigate the professional self-concept and incidence of burnout among medical school clinical faculty members, and to ascertain the factors that affect professional self-concept with respect to burnout.

**Methods:**

A total of 68 clinical faculty members at the Ajou University School of Medicine completed a modified form of the professional self-concept scale and the Maslach Burnout Inventory. We undertook the following statistical analyses: a descriptive analysis to understand the distribution of participants, correlation analysis to indicate associations between variables and a multiple regression analysis to examine the influence of gender, position, and specialty on professional self-concept and burnout, and the effect of each subscale of professional self-concept on burnout.

**Results:**

As professional self-concept increases, burnout decreases. There was no significant difference between professional self-concept and burnout with respect to gender or field of medical specialty, while a significant difference was observed across faculty position levels. Additionally, the professional self-concept subscale, which included satisfaction and communication skill, was found to significantly affect burnout.

**Conclusions:**

This study shows that professional self-concept affects burnout. Through these results, we can infer that professional self-concept functioned to protect medical school faculty from burnout. This may be a strategy that fortifies the professional identity of medical school faculty, and it is suggested that educational programs that are directed toward this goal be established.

## Background

Burnout in the workplace is characterized by emotional stress associated with work or with interpersonal relationships factors such as sustained response, leading to emotional exhaustion, depersonalization, and reduced personal accomplishment [1]. It has been shown that burnout is experienced more frequently by professionals employed in providing services to treat psychological, social, and physical problems [2]. Medical school faculty members have been reported to be highly likely to suffer burnout as a result of their job activities, including student education, research, clinical practice, and other medical administrative duties [3–6]. In particular, medical faculty who are also performing clinical duties are under pressure to increase their clinical productivity and, in many cases, are urged to treat patients rather than teach students [7, 8]. Therefore, there are numerous medical faculty members interested in student education who are unable to devote sufficient energy to teaching due to the lack of time and energy [9]. As a result, medical faculty who suffer from burnout also lose morale and experience both mental and physical problems [10].

The effects of burnout among medical faculty are not confined to the individual physician, but may result in the impairment of patient care [11], an increased incidence of medical errors and adverse events [12, 13], and decreased empathy towards patients [14]. Medical faculty burnout also has a detrimental effect on the education of medical students, as efforts to improve the quality of education need to be implemented; including enhanced interest in improving curricula and teaching methods. However, the ensuing physical and mental exhaustion that leads to the burnout of medical faculty may lead to passivity in accepting new and innovative changes [15, 16]. Therefore, rather than looking for a long-term solution to medical faulty burnout, we suggest that efforts to minimize burnout should be considered so that medical practice and medical education may be performed with a high level of quality.

The majority of medical school faculty are overburdened with their respective duties, but not all faculty experience burnout. There may be individual differences even within the same workplace environment in terms of how individuals view, interpret, and handle burnout. There has been increasing attention on the idea of professional self-concept, which reduces an individual’s burnout.

Professional self-concept is the perception of what it means to be and act as member of a profession [17]. More precisely, professional self-concept refers to the perceptions of a member of a specific group with his/her skill, knowledge, beliefs, values, and motivation, which are formed and changed in the process of evaluating his/her own traits and abilities through various experiences and interactions with others [18–21]. In other words, professional self-concepts are formed through self-reflection and social interaction. Professional self-concept affects an individual’s thoughts, behaviors, and emotions [22, 23]. Research in other areas suggests that individuals who have a strong, well-formed professional self-concept have low levels of burnout and are less willing to quit their jobs [18, 19]. On the other hand, those who do not have a well-formed professional self-concept tend to fail to function properly in their specialties and experience a high level of exhaustion or resignation without success [24].

Studies in many professions have shown that burnout influences professional self-concept in many professional workplaces. In a study of registered nurses in a university hospital setting, it was observed that nurses who participated in a professional identity development training program experienced an enhanced professional self-concept and lower levels of burnout [25]. Further, among professional school counselors, a negative correlation was found between professional self-concept and burnout [26].

In light of these results, it is our hypothesis that professional self-concept may be a moderating factor on burnout in medical school faculty members. However, there is a paucity of data on the relationship between professional self-concept and burnout in this highly specialized occupational group, and therefore, it is the aim of this study to delineate the role of professional self-concept in burnout in this group.

Our research questions are as follows:

- First, what is the extent/level of professional self-concept and burnout among medical school faculty members?
- Second, what are the effects of gender, job position, and medical specialty on professional self-concept and burnout in medical school faculty members?
- Third, what is the relationship between professional self-concept and burnout in medical school faculty members?

## Methods

### Participants

The participants of this study were employed clinical faculty members of Ajou University School of Medicine, Gyonggi Province, Republic of Korea. The questionnaire survey, conducted in April 2018, was sent as e-mails to 230 clinical faculty members who were engaged in teaching medical students in the 2017 academic year. Consent was obtained from all participants, and the questionnaire did not include the name of the participant. A total of 68 questionnaire replies was analyzed in this study.

### Measures

#### Professional self-concept

To measure professional self-concept, the scale for the professional self-concept of nurses developed by Arthur [27] was used in this study, as it is a scale that can evaluate various skills and attitudes. Although developed for the nursing profession, there are no items that would be inappropriate to apply to physicians. Only items where the term “nurse” or “nursing” is used were modified for physicians for use in this study. This professional self-concept scale consists of three subscales: professional practice, job satisfaction, and interpersonal communication. Professional practice included leadership, flexibility in adapting to one’s situation, and skills to ensure a competent work performance. Job satisfaction was defined as the degree of satisfaction and joy experienced in performing one’s job duties. Interpersonal communication refers to empathy and open-mindedness in interpersonal relationships. This instrument consists of 27 items: professional practice (16 items), satisfaction (7 items), and communication (4 items). As examples, professional practice is assessed through statements such as “Decision-making is one of my attributes,” “When I’m at work and the situation calls, I am able to think of alternatives,” and “I pride myself on my skills as a doctor.” Satisfaction is assessed through statements such as “Medicine is a rewarding profession.” Communication is assessed through statements such as “I don’t believe I am particularly empathic.” Each item was scored on a four-point Likert scale from “strongly do not agree” (one point) to “strongly agree” (four points), and a higher score was deemed to indicate higher levels of professional self-concept. To verify the reliability of this scale, we checked the Cronbach’s α coefficient, which was 0.925 in this study.

#### Burnout

To assess the degree of burnout among medical school faculty members, we employed the Maslach Burnout Inventory—Educators Survey (MBI-ES) that was developed by Maslach et al. [1]. This MBI is recognized as the leading measure of burnout and has been validated by extensive research conducted over a long period of time. This scale comprises 22 items that survey emotional exhaustion (EE, 9 items), depersonalization (DP, 5 items), and reduced personal accomplishment (RPA, 8 items). Each item in this study was measured on a seven-point rating scale ranging from “never” (zero points) to “always” (six points), and higher scores indicated more frequent burnout occurrences. Cronbach’s α for the scale was 0.905.

#### Analysis

To understand the distribution of participants by gender, job position, and job specialty, and to ascertain the characteristics of the questionnaire participants, descriptive statistical methods were applied. To identify the relationships between the subscales of variables, correlation analysis was conducted. Multiple regression analysis was carried out to analyze the effects of gender, faculty position, and field of specialty on professional self-concept and burnout. Multiple regression analysis was also applied to analyze the effects of the variables assessed by the professional self-concept subscales on burnout.

## Results

### Demographics

The distribution of gender, faculty position, and field of specialty of the study participants is shown in Table 1. Out of the 230 participants, 68 of them responded (29.6%), and the gender ratio was found to be 1.5 (M:F ratio). The distribution of faculty positions was fairly even, while the most common field of specialty was internal medicine, followed by surgery, and support care medicine.

**Table 1:** Characteristics of participants (*N* = 68)

Variable	*N*(%)
Gender	
Male	41 (60.3)
Female	27 (39.7)
Faculty Position	
Assistant professor	27 (39.7)
Associate professor	20 (29.4)
Professor	21 (30.9)
Field of Specialty	
Internal medicine	30 (44.1)
Surgery	21 (30.9)
Supportive care medicine^a^	17 (25.0)

^a^The Supportive care medicine field includes emergency medicine, rehabilitation medicine, anesthesiology, radiology, and clinical pathology

The analyses of participants’ professional self-concept and burnout are shown in Table 2.

**Table 2:** Means, standard errors, and standard deviations for professional self-concept and burnout

Variable	Mean	*SE*	*SD*
PSC	3.09	0.05	0.41
Professional practice	3.30	0.05	0.41
Leadership	3.10	0.07	0.54
Flexibility	3.37	0.05	0.42
Skill	3.44	0.05	0.38
Satisfaction	3.06	0.07	0.56
Communication	2.89	0.06	0.51
Burnout	2.39	0.10	0.85
EE	3.02	0.12	0.99
DP	2.01	0.14	1.14
RPA	2.16	0.11	0.94

*PSC* Professional Self-Concept, *EE* Emotional Exhaustion, *DP* Depersonalization, *RPA* Reduced Personal Accomplishment

Among the professional self-concept subscales, participants scored the highest on professional practice (*M* = 3.30*, SD =* 0.41), followed by satisfaction (*M* = 3.06, *SD* = 0.56), and communication (*M* = 2.89, *SD* = 0.51); while on the subscales of professional practice, participants scored the highest for skill (*M* = 3.44, *SD* = 0.38) followed by flexibility (*M* = 3.37, *SD* = 0.42), and leadership (*M* = 3.10, *SD* = 0.54).

Participants’ scores on the subscales for burnout were ranked as follows: emotional exhaustion (*M* = 3.02, *SD* = 0.99), reduced personal accomplishment (*M* = 2.16, *SD* = 0.94), and depersonalization (*M* = 2.01, *SD* = 1.14), in decreasing order.

The correlations between variables are shown in Table 3. It can be seen that there exists a statistically significant negative correlation (*r* = − 0.76, *p* < 0.01) between professional self-concept and burnout, indicating that higher professional self-concept values lead to lower burnout values. All the subscales of professional self-concept showed significant negative correlations with burnout, with their absolute values decreasing from 0.69 for satisfaction (*p* < 0.01) to 0.60 for communication (*p* < 0.01) and professional practice (*p* < 0.01).

**Table 3:** The relationship between professional self-concept and the burnout subscales

	Burnout	EE	DP	RPA
PSC	- 0.76^**^	- 0.59^**^	- 0.51^**^	- 0.83^**^
Professional practice	- 0.60^**^	- 0.40^**^	- 0.38^**^	- 0.74^**^
Leadership	- 0.63^**^	- 0.41^**^	- 0.42^**^	- 0.78^**^
Flexibility	- 0.56^**^	- 0.37^**^	- 0.36^**^	- 0.69^**^
Skill	- 0.41^**^	- 0.29^*^	- 0.24	- 0.52^**^
Satisfaction	- 0.69^**^	- 0.65^**^	- 0.43^**^	- 0.65^**^
Communication	- 0.60^**^	- 0.38^**^	- 0.46^**^	- 0.68^**^

^*^p < 0.05, ^**^p < 0.01

*PSC* Professional Self-Concept, *EE* Emotional Exhaustion, *DP* Depersonalization, *RPA* Reduced Personal Accomplishment

We investigated the effects of gender, faculty position, and field of specialty on professional self-concept and burnout as reported in Table 4. Gender and field of specialty were not found to be associated with professional self-concept and burnout, while faculty position did significantly affect them. Faculty position had a positive effect on professional self-concept, but a negative effect on burnout.

**Table 4:** Multiple regression analysis for professional self-concept and burnout

Dependent variable	Independent variable	Standardized Coefficients	*t*	*R* ^*2*^	*adj. R* ^*2*^	*F*
		*β*				
PSC				0.13	0.09	3.17
	Gender	- 0.03	−0.21			
	Position	0.35	2.91^**^			
	Specialty	- 0.10	- 0.83			
Burnout				0.29	0.25	8.50
	Gender	0.05	0.46			
	Position	- 0.51	−4.70^***^			
	Specialty	- 0.08	- 0.74			

^**^p < 0.01, ^***^p < 0.001

*PSC* Professional Self-Concept

The analysis of the effect of professional self-concept on burnout is shown in Table 5. Professional self-concept explained 60% of the variance (*F* = 30.13, *p* < 0.001) for burnout. It was also observed that while professional practice did not have a significant effect on burnout, satisfaction (*β* = − 0.41, *p* < 0.001) and communication (*β* = − 0.30, *p* < 0.01) had significant negative effects.

**Table 5:** Multiple regression analysis for burnout

Variable	Unstandardized Coefficients		Standardized Coefficients	*t*
	*Β*	Std. Error	*β*	
Professional practice	−0.43	0.22	−0.21	−1.98
Satisfaction	−0.62	0.16	−0.41	−3.85^***^
Communication	−0.50	0.16	−0.30	−3.13^**^
*R*^*2*^ = 0.59, adj. *R*^*2*^ = 0.57, *F* = 30.13^***^				

^**^p < 0.01, ^***^p < 0.001

## Discussion

This study investigated the effect of professional self-concept on burnout in medical school clinical faculty members. The study results are as follows.

First, the analysis of the relationship between professional self-concept and burnout found negative correlations between professional self-concept and all burnout subscales, emotional exhaustion, depersonalization, and reduced personal accomplishment. That is, medical faculty with higher levels of professional self-concept experience less burnout. Our results can be understood within the framework of Edwards and Dirette [28], whose study demonstrated a significant negative correlation between occupational therapists’ professional self-concept and burnout. Our results confirm that strengthening the level of professional self-concept could lead to decreases in burnout occurrences. In particular, the results show that professional self-concept is strongly and positively related to the personal accomplishment sub-scale of the MBI. Hence professional self-concept appears to be strongly influenced by experiencing a sense of competence and accomplishment in one’s field of expertise.

Second, it was found that the higher the faculty position, the higher the level of professional self-concept with accompanying lower levels of burnout. This implies that junior faculty members experience lower levels of professional self-concept than senior faculty members, and have concomitantly higher levels of burnout. Earlier investigations of medical school faculty burnout [29–31], concluded that in their early career years, faculty were more susceptible to high levels of emotional exhaustion, which agrees with our results. The reason senior faculty have less burnout than junior faculty may be understood in terms of accumulated career experience, which is accompanied by enhanced job skills and control abilities so that high standards of competence are achieved, leading to relatively decreased job-related stress or burnout [29, 32]. In addition, junior faculty members work longer hours than senior faculty members, receive more emergency calls, and are subject to irregular work-life problems [33, 34]. Family environment may also be a contributing factor for burnout. Junior faculty are younger and frequently have families with young children to support [30], which may be contributing factors to conflict and burnout in the workplace and at home.

Third, while the subscales of professional self-concepts, such as leadership, flexibility, and skill did not have a significant effect on burnout, our study found that job satisfaction and communication factors had a significant negative effect. Medical school faculty form various interpersonal networks with patients, medical students, nurses, and administrative staffs in two workplace environments—at hospital and at school. The difficulty in interpersonal communication is a factor that elevates the degree of burnout. It was shown in an earlier analytic study of the relationship between burnout and job satisfaction in surgeons that lower job satisfaction was correlated with a significantly greater incidence of burnout [9]. Another study addressed the relationship between registered nurses’ training in communication skills and burnout, and determined that communication skill training played an active role in ameliorating burnout among nurses [35]. We were therefore able to ascertain from the aforementioned studies whether the extent of medical school faculty satisfaction with their duties and how well they are able to communicate with whom they interact in their workplace significantly affect their levels of burnout.

The significance and implications of this study are as follows.

First, the main point of the current study is that we were able to explore the relationship between professional self-concept, and confirm the function of professional self-concept in ameliorating this burnout. The findings of the study do not necessarily demonstrate a causal relationship between professional self-concept and burnout. Nonetheless, it may be considered that professional self-concept is a possible factor serving a protective function for the medical faculty member’s mental well-being. In particular, it is our opinion that one of the major findings of this explorative inquiry is the confirmation of the significant role of job satisfaction and interpersonal communication skills in reducing the degree of burnout among medical faculty.

Second, we would point out that junior medical faculty have low levels of professional self-concept and high burnout rates. To enhance their professional self-concept levels and decrease burnout rates, junior medical faculty need faculty development and mentoring programs. We suggest that these results be brought to the attention of medical school deans and administrations so that necessary institutional support can be provided.

Nevertheless, we must note the limitations of the current study to suggest directions for future follow-up studies. We find the distribution of gender, position, and specialty to be relatively balanced, even with a small number of participants. In particular, we think the 6:4 gender ratio in this study is not terribly important. Nevertheless, this study has limitations in that it was conducted in one medical school with a small sample. It is necessary to conduct follow-up studies in more number of medical schools with larger samples. Moreover, this study applied a standardized questionnaire that assessed self-reported measures; other standardized instruments could also be used advantageously. Professional self-concept is one aspect of the educational process that gradually develops through adaptation to job duties, and therefore requires continuous evaluation at each step over a long period. There is also a limitation in the overall measurement of burnout, and the use of quantitative instruments to measure the psychodynamic aspects of this psychological phenomena. To better understand the burnout phenomenon, in-depth interviews along with qualitative research is deemed imperative.

## Conclusions

This study ascertained that professional self-concept functions to protect medical school faculty from burnout. This may be a strategy that fortifies the professional identity of medical school faculty, and it is suggested that educational programs directed toward this goal be established.

## Data Availability

The dataset used during the current study is available from the corresponding author upon reasonable request.

## Abbreviations

DP: Depersonalization
EE: Emotional Exhaustion
MBI-ES: Maslach Burnout Inventory-Educators Survey
PSC: Professional Self-Concept
RPA: Reduced Personal Accomplishment
